# Vitreomacular interface abnormalities in the Ghanaian African

**DOI:** 10.1038/s41433-023-02737-z

**Published:** 2023-09-29

**Authors:** Winfried M. Amoaku, Laura Cushley, Vittorio Silvestri, Stephen Akafo, Kwesi N. Amissah-Arthur, Seth Lartey, Courtney N. Hageman, Christian M. Pappas, William C. Hubbard, Paul S. Bernstein, Albert Vitale, Megan Roberts, Gianni Virgili, Gregory S. Hageman, Giuliana Silvestri, For the Ghana AMD Study Group, Edem K. Ahiabor, Amos Atkins, Jessica Feilmeier, Michael Feilmeier, Lisa S. Hancox, Sheri L. McCormick, Norma Miller, Lisa R. Nichols, Laura N. Cushley, Cóilin P. Ferrin, Stacie Matthews, Susie Choi, Christopher Ricks

**Affiliations:** 1 Academic Ophthalmology, Mental Health & Clinical Neurosciences, University of Nottingham and University Hospitals, Nottingham, UK; 2 Centre for Public Health, Queen’s University of Belfast, Belfast, UK; 3 NICRN, Belfast Health & Social Care Trust, Belfast, UK; 4 Unit of Ophthalmology, Department of Surgery, University of Ghana Medical School, Korle Bu, Accra, Ghana; 5 Eye Unit, Eye Ear Nose and Throat Department, Komfo Anokye Teaching Hospital and Kwame Nkrumah University of Science and Technology, Kumasi, Ghana; 6 Department of Ophthalmology & Visual Sciences, Moran Eye Center, Sharon Eccles Steele Center for Translational Medicine, University of Utah, Salt Lake City, UT USA; 7 Ophthalmology Services, Eye and ENT Clinic, Royal Victoria Hospital, Grosvenor Road, Belfast, BT12 6BA UK; 8 Department of Microbiology & Immunology, University of Iowa, Iowa city, IA USA; 9 Department of Ophthalmology, University of Iowa, Iowa city, IA USA

**Keywords:** Retinal diseases, Vitreous detachment

## Abstract

**Background/Objective:**

Describe vitreomacular interface abnormalities (VMIA) using spectral-domain optical coherence tomography (SD-OCT), and correlations with age-related macular degeneration (AMD) grade in Ghanaian Africans.

**Subjects/Methods:**

Prospective, cross-sectional study of adults aged ≥50 years recruited in Ghana AMD Study. Participant demographics, medical histories, ophthalmic examination, digital colour fundus photography (CFP) were obtained. High-resolution five-line raster OCT, Macular Cube 512 × 128 scans, and additional line scans in areas of clinical abnormality, were acquired. SD-OCT VMI features classified by International Vitreomacular Traction Study Group system and relationships to AMD grade were evaluated. Outcomes: VMIA prevalence, posterior vitreous detachment (PVD), vitreomacular adhesions (VMA), vitreomacular traction (VMT), epiretinal membranes (ERM), correlations with AMD grade.

**Results:**

The full Ghana AMD cohort included 718 participants; 624 participants (1248 eyes) aged ≥50 years (range = 50–101, mean = 68.8), 68.9% female were included in this analysis. CFP with OCT scans were available for 776 eyes (397 participants); 707 (91.1%) had gradable CFP and OCT scans for both AMD and VMI grading forming the dataset for this report. PVD was absent in 504 (71.3%); partial and complete PVD occurred in 16.7% and 12.0% respectively. PVD did not increase with age (*p* = 0.720). VMIA without traction and macular holes were observed in 12.2% of eyes; 87.8% had no abnormalities. VMIA was not significantly correlated with AMD grade (*p* = 0.819).

**Conclusions:**

This provides the first assessment of VMIA in Ghanaian Africans. VMIA are common in Africans; PVD may be less common than in Caucasians. There was no significant association of AMD grade with VMIA.

## Introduction

Evidence to date suggests that age-related changes in the vitreous are likely to have significant impact on the pathogenesis of macular diseases and function [1–8]. Adhesions between the internal limiting membrane (ILM) and the vitreous become less robust with age [7, 9, 10]. This, in addition to vitreous liquefaction [5], results in posterior vitreous detachment (PVD) which usually initiates at the macula, but may occur simultaneously at multiple sites [11–13]. PVD begins to occur at approximately 40 years of age in Caucasians, although the process may occur earlier in eyes with high myopia, or other posterior segment diseases [1, 5]. It may develop over several years, rather than instantaneously as one sudden event [12, 14]. It is further suggested that vitreomacular traction (VMT) results from partial PVD, with peripheral separation in association with adherence in the macula and optic nerve, of the vitreous [4, 6, 15]. VMT is thought to develop as a result of proliferation of fibroblasts on the surface of persistent cortical vitreous attachments to the ILM [10, 16, 17]. This concept is supported by the clinicopathological study by Gandorfer et al. [18] who reported two types of VMT syndrome with different forms of epiretinal fibrocellular (myofibroblasts and astrocytes) proliferation and collagen deposition. Chang et al. have reported similar findings [19].

Evaluation of the vitreoretinal interface (VMI) has been significantly enhanced by the recent introduction of optical coherence tomography (OCT) (especially high-resolution OCT), into clinical practice [20–26]. It is non-invasive and considered a valuable tool for evaluating the VMI, including vitreomacular adhesions (VMA) or VMT detection and configuration analysis [19]. An international systematic clinical grading system of VMI pathology based on OCT imaging was developed recently [2].

Several VMI studies in Caucasian populations exist and indicate that variations in the strength of VMA lead to varying risks in PVD and potential complications [11, 12]. Similarly, the invariable association of VMT and epiretinal membranes (ERM) have been described [8, 27–29]. The Beaver Dam Eye Study (BDES) recently reported the population prevalence of VMI abnormalities (VMIA) in participants aged 63 years and older [29]. McKibbin et al. [30] reported on VMI grading on OCT in 25% of 8359 (2090) participants with visual impairment (VI) (in at least one eye) in the UK Biobank study (aged 54–66 years), which included 374 (4.9%), 73 (4.4%), and 22 (5.3%) Black or Black British controls without VI, participants with VI without vitreoretinal interface abnormalities (VRIA), and those with VI and VRIA, respectively [30].

Other studies have implicated VMT in the causation or prognosis of various diseases including age-related macular degeneration (AMD) [31–37], and diabetic macular oedema (DMO) [14, 38–42]. A high incidence of VMT has been noted in treated eyes after photodynamic therapy [43]. More recently, VMA was suggested to be a prognostic factor in the visual outcomes following intravitreal injections of anti-VEGFs [44–47]. Krebs et al. [27] reported that the spectral-domain OCT showed a high prevalence of type III neovascular AMD lesions in eyes with VMA in the macula. A subsequent study by Waldstein et al. [48], however, reported that VMAs do not influence the development of exudative AMD, or time to progression. Jackson et al. [49] undertook a meta-analysis of vitreous attachments in eyes with AMD, DMO and RVO, and reported that eyes with nAMD were twice as likely to develop VMA, and less likely to have PVD. However, Maggio et al. [6] concluded from their study, which included 1067 eyes that there was no significant difference in VMA prevalence in AMD eyes compared with age-matched controls, and further, that there was no difference in developing de novo choroidal neovascularisation (CNV) in eyes with or without VMA. They further suggested that VMA may be a consequence of CNV development. Other studies have described VMA abnormalities in eyes with DMO undergoing laser photocoagulation [50].

More recently, the Handan study reported on the prevalence of ERM in a rural Chinese adult population [51], and the MONTRACHET study reported a high prevalence of VMIA in French (Caucasian) participants aged 75 years and older [52]. Similarly, a prevalence of 15.9% VMI disorders in a south Netherlands (mainly Caucasian) population of 40–75 years olds, which was related to age, sex and glucose metabolic status was reported in the Maastricht study [53].

Previous reference to racial differences in the vitreoretinal interface (VRI) was made by Av-Shalom [54] who suggested that degenerative vitreous changes were rare in Africans compared to Caucasians. To the best of our knowledge, only one report exists on VMI changes and potential associations with AMD in the north-eastern African population, from Egypt [3], which found no association between VMA and AMD [3]. The systematic review by Xiao et al. [55] confirmed the lack of studies on the VMI from Africa. As such, there are only limited data on the occurrence of VMT in the African. In particular, there are no studies on VMI from sub-Saharan Africa. The recent ultrastructural correlations of fibrocellular proliferations along the exposed inner retinal and posterior vitreous surfaces correlating with pre-operative OCT findings in eyes with VMT may have significant implications for different populations [21]. As collagen formation and gliosis may be different in the African compared to other races, an evaluation of the vitreoretinal VRI in such populations may help to elucidate the role of the VMI in vitreoretinal disease pathogenesis.

We hypothesized that VMIA occurs more frequently and is more adhesive in the Ghanaian population in the age group ≥50 years. We evaluated the VMI in all available OCTs of the macula obtained from patients in a Ghanaian population as part of the AMD study in Ghana (The Ghana AMD Study) which included participants with retinal diseases and controls. We investigated any potential association of the VMI status with the AMD grade in each eye.

The purpose of this report is to document changes at the VMI with spectral-domain (SD) OCT in this previously unreported Ghanaian African population and to investigate any association with AMD occurrence.

## Subjects and methods

### Study population

Participants in this study were recruited as part of the larger ‘Ghana AMD Study’, a prospective, cross-sectional study designed to investigate AMD in the Ghanaian population. The study was conducted in two teaching hospitals, Korle-Bu Hospital (KBTH), Accra, and Komfo Anokye Teaching Hospital (KATH), Kumasi, during five different visits: March 2009 (Accra), April 2010 (Accra and Kumasi), February 2012 (Accra), April 2017 and January-February 2020 (Accra). The study enroled and consented Ghanaians aged 50 years and older. The research adhered to the Tenets of the Declaration of Helsinki and the protocol was approved by the institutional Ethics Review Boards of both KBTH (University of Ghana Medical School Protocol ID No: MS-Et/M.4-P./2008/2009) and KATH (Reference: KNUST CHRPE/32/10). Neither the study individuals nor the public were involved in the design, conduct, reporting, or dissemination plans of this study.

### Ghana AMD study

Study details were explained to all study subjects with the aid of interpreters; participant information leaflets were provided and written consent obtained. All patients enroled were of Ghanaian ethnicity. A questionnaire detailing the participant’s demographic details, ophthalmic and medical histories, was completed by local ophthalmic nursing staff. Best-corrected Snellen visual acuity with glasses or pinhole was assessed, intraocular pressures were measured with a Goldmann applanation tonometry, and the pupils were dilated using tropicamide ophthalmic 1% and phenylephrine ophthalmic 2.5% solutions. Refraction was not assessed. Each patient had slit lamp biomicroscopy and fundus examination by a retinal specialist. Digital colour fundus photographs were captured using the Canon CR-DGI Non-Mydriatic Camera. Fields F1 (optic disc centred) and F2 (macula-centred) were captured in stereo. Peripheral images of any abnormalities noted on fundus examination were captured when necessary. A subset of patients was also imaged using a Zeiss Cirrus HD-OCT, Model 400 (on the second and third visits) or Topcon 3D-OCT 2000 on the fourth study trip as the Cirrus was no longer functional. Retinal images were graded (AV, GS, PSB, WA) and the participant’s eyes were classified according to their retinal status. AMD grading was independently performed by three masked investigators with retinal expertise (GS, GSH and WMA) based upon the Rotterdam AMD classification [56], as previously described in our manuscript on West African Crystalline Maculopathy [57]. When there was a disagreement, panel adjudication involving all three investigators was used to derive consensus. The presence of cataract and glaucoma were recorded in the grading information but was not used for classification for the purpose of this report.

SD-OCT imaging was available for the second, third and fourth study trips, which provided the opportunity to assess OCT features of normal and diseased eyes in this population. As such, participants recruited on the first and fifth trips were excluded from this analysis. The Cirrus HD-OCT 400 scanner was originally selected because of the advantage that the technique of obtaining images do not rely on the patient’s ability to fixate, and that the data can be reviewed later and the position of the scan relocated to the centre of the fovea. This was important as we anticipated that due to medial opacities in this population, scanning might be difficult. The scan pattern selected was composed of high-resolution five-line raster scans and the Macular Cube 512 × 128 scan. If any abnormality was noted on clinical examination, additional scans were performed in the relevant areas. On the fourth trip, when the Cirrus was not functional, OCT images were obtained with the Topcon 3D-OCT 2000. Macular thickness was measured using the Cirrus HD-OCT and the Topcon 3D-OCT 2000 generated thickness maps using the Cirrus Macular Thickness Analysis and Topcon ImageNet tools.

### OCT image grading

All eyes with gradable scans were included in this study. Both eyes in any individual, when available, were included as changes in the vitreoretinal interface are not necessarily symmetrical in any single individual. In eyes where the OCT signal strength was reduced below four, or where fovea or optic disc were not visible, scans were considered ungradable and the scans excluded from further analyses.

The present report focuses on eyes with and without pathology with particular reference to eyes with AMD. Eyes that had gradable OCTs but had confounding retinal vascular pathology in the macular area causing disruption the inner retina, were excluded.

The VMI was assessed by a certified retinal grader (LNC) with and adjudicated by retinal experts (GS, GSH, WMA). OCT scans were assessed at different sittings using all the available OCTs as described by Ghazi et al. [38], and the International Vitreomacular Traction Study Group (IVTS) classification as summarized in Table 1 [2]. A panel adjudication involving all three retinal experts was used to derive consensus where there was disagreement. VMA was defined as cortical vitreous detachment in the perifoveal region, but with attachment within the 3 mm radius of the fovea (i.e. within the 3 ETDRS circles) without alteration within the foveal contour (or underlying retinal tissue). If attachment of the hyaloid membrane over the fovea/macula was at a shallow angle, *i.e*. without a steep angle of attachment or focal distortions of the retina, VMA was further subdivided according to the width of attachment, as to whether the attachment was narrow/focal based or broad based.

**Table 1:** Definitions of vitreomacular adhesion (VMA), traction (VMT) and macular hole (MH).

Classification	Subclassification
Vitreomacular adhesion (VMA)	Size: focal (<1500 mm) or broad (>1500 mm) Isolated or concurrent
Vitreomacular Traction (VMT)	VMTFT Size: focal (<1500 mm) or VMTBT broad (>1500 mm); Isolated or concurrent
Full-thickness macular hole (FTMH)	Size: small (<250 mm), medium (>250 to <400 mm), or large (>400 mm) Status of vitreous: with or without VMT Cause: primary or secondary

The International Vitreomacular Traction Study Classification System for Vitreomacular Adhesion, Traction, and Macular Hole [2].

*VMTFT* focal vitreomacular traction, *VMTBT* broad vitreomacular traction.

VMT was diagnosed when there was evidence of perifoveal vitreous detachment from the retinal surface within the 3 mm radius of the fovea, where there were structural change in the fovea and a steep slope of the posterior hyaloid membrane to the inner macular surface. In addition, VMT was present when there was a sharp angulation of the hyaloid, and/or localized deformation of the retinal profile detectable at the adhesion site of the posterior hyaloid membrane. VMT was divided into focal (VMTFT), and broad vitreomacular traction (VMTBT). The presence of focal horizontal attachments of the posterior hyaloid membrane to the macula in more than one location was described as tangential or horizontal VMT (VMTHT). Defects in the inner fovea with preservation of the outer foveal layers including photoreceptors is referred to as lamellar macular hole, whilst loss of all foveal retinal layers was graded as full-thickness macular hole.

The presence of associated macular distortions or oedema, ERM (focal thickening of the hyaloid membrane on the surface of the retina) which manifested as focal hyper-reflective signal on the inner surface of the retina, or other associated findings were noted. Complete separation of the hyaloid membrane from the macular area indicated by a clear visible posterior vitreous cortex, which moved freely in the vitreous cavity was considered as a complete PVD. Care was taken to distinguishing this from simulations of complete PVD by the presence of a liquid-filled pre-retinal bursa, in the presence of persistence of a pre-macular vitreous cortex [11, 16, 58]. When the pre-macular bursa is visible, but the vitreous cortex is not detected, then PVD was absent. Persistent attachments to the optic disc margins were also assessed in eyes that had PVD. Eyes with retinal vascular disease were classified as ‘concurrent’ as per IVTS classification [2].

The results are reported in terms of the presence or absence of PVD and whether this was complete or partial; the presence and type of VMIAs including adhesions. These changes were correlated with AMD grade or co-existing macular disease.

Data were entered into IBM SPSS Version 25 predictive analytics software. The data were cleaned and a random 10% data entry recheck was carried out.

### Statistical analyses

Descriptive statistics were used to summarize the occurrence of VMIA and associated diagnoses. All eyes in participants aged 50 and over which were both gradable for both VMIA (absent, present) and AMD (none, early, GA, neovascular AMD) were examined for relationship between VMIA and AMD, together with odds ratios (OR) (and 95% confidence intervals) measuring risk of VMIA (+ve) for stages of AMD relative to no AMD.

It should be noted that most participants provided two eyes in the study and those two eyes were not independent observations. Therefore, we conducted statistical analyses accounting for within-subject correlation using mixed models (linear or logistic as appropriate), with individuals as random effects. The software Stata 17.0 (StataCorp, College Station, TX) was used to calculate OR and 95% CIs.

## Results

### Total AMD study cohort demographics

A total of 1436 eyes of 718 participants were examined during the first four visits for the total cohort. The demographics of the total cohort were as follows: age range was 22–101 years, 65.9% were female and 32.9% male, mean age was 65.0 years. The mean age of the females was 67.1 years (range 22–101), and of the males 69.9 years (range 50–100).

Of the total participants recruited, 624 participants (1248 eyes) were aged over 50 years and were included in the analyses. Participants aged 50 years and older had a mean age of 68.79 ± 10.21 (range 50–101) years and included 388 male eyes (31.1%) and 860 female eyes (68.9%). Colour fundus images were available on 1414 of the total 1436 eyes (98.5%), 92.6% of which were gradable. OCT scans were available for 891 of the total 1436 eyes (62.0%). OCT scans from 36 eyes were ungradable due to media opacity. Gradable OCT scans were available for 855 (59.5%) of eyes in the total cohort. Colour images and OCT scans were available for 776 eyes of 496 participants. Of the 776 eyes, 707 (91.1%) had gradable images and OCT scans for both AMD and VMI. These formed the dataset analyzed for this part of the project.

### VMI analysis—cohort demographics

The VMI dataset included 776 eyes, 505 female eyes (65.1%) and 271 male eyes (34.9%). The mean age of the females in the VMI analysis cohort was 67.1 years (range 50–100), and of the males 69.9 years (range 50–100). Gradable images and OCT scans for both AMD and VMI were available in 707 (91.1%) eyes. There were four eyes with non-proliferative diabetic retinopathy (NPDR), one of which was excluded due to the presence of cystoid macular oedema (CMO); two of the gradable eyes had signs of previous branch retinal vein occlusion (BRVO), but none had macular oedema or retinal disruption on OCT and so were included.

The demographic details (age–sex distribution) of patients who had OCTs were not different from the total study population (*p* = 0.57). In the cohort of 707 fully gradable eyes, 621 (87.8%) were found to have VMIA.

### Posterior Vitreous Detachment

PVD (partial or full) in this population was observed in 203 (28.7%) of 707 eyes, of which 118 (16.7%) had partial PVD and 85 (12.0%) had complete PVD. No PVD was observed in 504 eyes (71.3%). The occurrence of PVD was 40.1% (24% partial and 16.1% complete) at age >65 years and in 34.2% (18.4% partial and 15.8% complete) in those aged >80 years. The presence of PVD did not increase with age (*p* = 0.720 for linear trend).

Surface retinal disruption was present in 5.2% of eyes and an ERM was present in 13.2% of eyes. CMO was present in 3.3% of eyes. In eyes with no evidence of AMD (Stage 0 AMD) PVD was observed in 29 of 62 (46.8%) eyes. PVD occurred in five of 19 (26.3%) with neovascular AMD (Grade 4b) compared with 33 of 64 (48.43%) eyes with non-neovascular AMD. In eyes with geographic atrophy (AMD Grade 4a), the incidence of PVD was 25%. The findings are summarized in Table 2. The presence of PVD either partial or complete did not correlate with AMD grade (*p* = 0.93).

**Table 2:** Relationship between vitreomacular interface abnormalities (VMIA) and age-related macular degeneration (AMD).

	No VMIA	VMIA (any)	OR^a^	95% CI
No AMD	63	438	1.00	
Early AMD (Grades 1–3)	13	123	1.49	0.56–3.98
AMD Grade 4a (Geographic Atrophy)	3	21	1.43	0.18–11.6
AMD Grade 4b (Neovascular AMD)	7	39	0.82	0.22–3.02

^a^Accounting for within-subject correlation in a logistic mixed model.

### Vitreomacular interface abnormalities

VMIA was observed in 86 (12.2%) of 707 fully gradable eyes. VMA without traction (VMAWOT) was noted in 81 (11.4%) of eyes, VMTHT in seven (0.99%) eyes, VMTFT in 10 (1.4%) eyes, all of which were in eyes without any evidence of AMD or had Grade 1 AMD only. No eyes with neovascular AMD had associated VMTFT. VMTBT was uncommon, occurring in only 1 (0.1%) of eyes. Macular holes were observed in 4 (0.6%) of eyes, none of which had AMD. Overall, there was no significant correlation between the presence or absence of VMI abnormalities and AMD grade (*p* = 0.85).

CMO occurred in 3.1% of eyes. The oedema was associated with venous occlusions, exudative AMD, diabetes and ERM. ERM was noticeable in 50 (7.7%) of eyes. The retinal interface was disrupted in 4.7% of eyes.

### Relationship of VMI abnormalities to AMD

Table 2 shows the relationship between VMIA and AMD, together with odds ratios (and 95% confidence intervals) measuring risk of VMIA (+ve) for stages of AMD relative to no AMD. There was no evidence of an association between VMIA and AMD grading overall (*p* = 0.819), as well as for any AMD grade vs no AMD.

The foveal architecture was comparable to those reported in Caucasians. However, a gentler slope to the foveal pit that differed significantly in width, slope, and volume, was observed in the absence of any VMI abnormalities in this Ghanaian cohort.

## Discussion

It is known that, despite biomicroscopically visible PVD, VMA may persist. Similarly, biomicroscopy may not identify partial PVD [11, 12, 23, 27, 59]. OCT has recently superseded ultrasonography in evaluating the VMI has reported the SD-OCT was of higher precision in the evaluation of vitreoretinal adhesions, particularly in exudative AMD [12, 13, 19, 27, 37, 60, 61]. Larsson and Oesterlin [62] reported a 30% rate of complete PVD in individuals of 65 years or older, whilst Hayreh and Jonas [63] reported that PVD may be found in approximately two-thirds of this age group. Perichon et al. [61] showed that PVD was not detected in 25% of patients at 80 years or older; this was supported by a later study [37]. There have been several recent reports on OCT studies of the VRI in various retinal diseases including DMO, and diabetic traction macular changes [14, 23, 25, 39, 40]. Koizumi et al. [64] reported on the evaluation of the VRI with regards to VMT and ERM using SD-OCT [64]. However, only one study has been reported from a north-eastern African population (Egypt), and only in persons with different types of AMD [3]. This study is the first report on the VMI from a sub-Saharan African population with or without AMD.

In an OCT study conducted by Uchino et al. [13], no PVD was detected in 29.2%, whilst partial PVD of different grades occurred in 62.2%, and total PVD was observed in only 8.6% of eyes in a cohort of 31–74 years [mean 52.3 yrs]. The onset of PVD (in the Uchino series) began in subjects of less than 50 years who developed asymptomatic PVD localized to the perifoveal area and increased with age. Kumagai et al. [65] evaluated the VMI with OCT in eyes aged 22–95 years with various vitreoretinal diseases and reported vitreoretinal adhesions in 15% of healthy eyes (average age 66.4 ± 12.7 years), as compared to 9% in eyes with AMD (average age 68.0 ± 12.3 years). They also reported that vitreoretinal traction occurred in 2% of healthy eyes compared to 1% in eyes with AMD. These differences were statistically significant. Kumagai et al. [65], however, did not describe any variations of these changes with age.

In this study in the Ghanaian population, the 40.1% occurrence of PVD at age >65 years and in 34.2% in those aged >80 years is significantly less than that reported from previous studies with ultrasonography in other populations. Larsson and Osterlin [62] reported a complete PVD rate of 30% in those aged 65 years and older, which is significantly higher than the 16.1% (95% CI 12.3–20.7) found in the Ghanaian population in this study. Hayreh and Jonas [63] reported a total PVD rate of 60% in those aged ≥65 years, again, significantly different from that in our study population. Perichon et al. [61] reported a PVD rate in those aged 80 years and over in 75% of individuals which contrasts with a rate of 34.2% (95% CI 24.5–45.4) in the Ghanaian population. Explained differently, approximately 60% of eyes of patients >65 years, and 65% of >80 years in our study did not have any detectable PVD on OCT.

The Beijing Eye study which included persons aged 50–93 years old, reported the population prevalence of incomplete PVD was 60.5% of eyes, and was associated with younger age, and male gender [66]. The Handan Eye Study reported a PVD prevalence of 2.7% associated with age in both women more than men in the population of 30 years and older (mean age = 52 years) [67]. The BDES recently reported the population frequency of VMI changes [29]. The BMES reported findings, similar to those in the BDES [59], whilst the NICOLA Study which included younger participants (mean age = 62 years) reported lower prevalences of VMA (22.6%), VMT (0.5%), ERM (7.6%), and macular holes (0.3%) [68]. Similarly, the UK Biobank study reported a 3.6% prevalence of VMI changes (in 55–66 year olds), with increasing age, female sex and Asian ethnicity as significant associations [30]. The study by Zapata et al. [69] included participants aged 45 years and older and reported a lower prevalence of vitreoretinal interface abnormalities [70].

The Maastricht study (2008), using OCT, reported that VMIA were associated with older age, female sex and glucose metabolic status [53]. The MONTRACHET study reported a high prevalence of VMIA in participants aged 75 years and older, including VMA (17.7%), VMT (1.4%), lamellar macular holes (1.0%), full-thickness macular holes (0.2%), macular pseudoholes (0.4%), epiretinal membranes (38.9%), and macular cysts (5.8%).^70^ VMA were associated with male sex, and negatively correlated with age and prior cataract surgery in that study, whilst older age and previous cataract surgery were associated with ERM [71].

It has been suggested that vitreous remnants on the retinal surface following PVD may predispose to epiretinal gliosis [10, 23, 60], and macular holes [16, 72]. In addition, worsening of proliferative diabetic retinopathy [60] and diabetic epiretinal gliosis [16, 72], which may induce macular oedema with further reduction in vision have been postulated as due to PVD. Sebag & Balazs [68] and Kumagai et al. [65] have shown that there was residual foveal deformation in eyes with macular PVD in patients with contralateral macular hole suggesting that pre-existing strong VMA precedes macular PVD in these eyes [69]. Doi et al. [70] previously described firm adhesions of the vitreous in the central macula [73]. As there was a high rate of vitreoretinal interface abnormalities in our cohort, it is possible that some of the visual deficit in our study population may be corrected by removal of persistent vitreous attachments and or ERMs. If such VMA or remnants are confirmed as increasing risk for reduced vision, pharmacologic vitreolysis, which is now on the horizon, will be useful as treatment.

In our study, it is significant that tangential traction in the foveomacular zone was found mainly in eyes with PVD. This finding supports the concept that incomplete PVD predisposes to VMT. It would therefore become more important that in patients with symptoms of visual impairment where no other clinical findings are obvious that the persistence of VMA in the posterior pole, despite PVD elsewhere, should be looked for [11, 58].

Previous studies have suggested that VMA may be important in the development of neovascular AMD [31–37, 45]. By implication, eyes (or populations) which have less PVD or more VMA should have a higher incidence/prevalence of neovascular AMD. In the present study, there is no association of VMA or VMT and neovascular AMD or geographic atrophy. The less frequent occurrence of neovascular AMD in this population compared to Caucasian populations would imply that VMA and VMT as such are not important in the development and/or progression of advanced AMD. This supports the studies by Waldstein et al. [48] and Maggio et al. [6] which concluded that VMA had no significant influence on the development or progression of neovascular AMD. It has also been suggested that the presence of VMT may affect the outcomes of treatment of neovascular AMD. Schmidt et al. [43] and Edwards et al. [74] surmised that VMT does not predispose to, but rather may be protective of AMD. They explained this by a referral bias in which patients with AMD and VMT are not referred for VMT surgery.

Differences in the literature regarding occurrence of VMIA are most likely due to the varied definitions adopted in the different studies. However, irrespective of such differences, this study confirms the value of OCT in evaluating the VMI.

This study provides the first report on the VMI in the Sub-Saharan African. The results suggest that PVD may be less common than in the Caucasian, and that vitreoretinal adhesions are common in Africans, and may be responsible for visual loss in this population. The results of this study do not support a role of VMIA in AMD.

The main limitation of this study is that the subjects may be self-selected as it is a hospital based, rather than a true population cohort (as we did not sample a representative population), and correlations of OCT findings with ultrasonography were not undertaken. Furthermore, it did not specifically obtain best-corrected vision, but rather the best habitual vision. It is therefore impossible to fully attribute visual symptoms to VMI abnormalities. As this study was based in Ghana, no data was obtained from Caucasians simultaneously under similar study conditions.

## Summary

### What is known before

- Age-related changes in the vitreous are likely to have significant impact on the pathogenesis of macular diseases and function.
- Evaluation of the vitreoretinal interface (VMI) has been significantly enhanced by the introduction of OCT.
- There are no studies on VMI from a sub-Saharan Africa population.

### What this study adds

- This study provides the first assessment of VMIA in the Ghanaian African.
- It suggests that VMIA, including adhesions with or without traction, are common in Africans and that PVD may be less common than in Caucasians.
- There was no association of AMD grade with VMIA.

## Data Availability

The data is available from the corresponding author upon reasonable request.
